# The nutritional status of children in Bhutan: results from the 2008 National nutrition survey and trends over time

**DOI:** 10.1186/1471-2431-12-151

**Published:** 2012-09-19

**Authors:** Ugyen Zangmo, Mercedes de Onis, Tandin Dorji

**Affiliations:** 1 Department of Public Health, Ministry of Health, Thimphu, Bhutan; 2 Department of Nutrition for Health and Development, World Health Organization, 20 Avenue Appia, Geneva 27, 1211, Switzerland

**Keywords:** Stunting, Wasting, Malnutrition, Infant nutrition, Child health

## Abstract

**Background:**

There are few reports on the nutritional status of Bhutanese children. The objective of this paper is to summarize results from the 2008 National Nutrition Survey and to describe progress achieved during the last two decades.

**Methods:**

A cross-sectional survey of 2376 children aged 6 to 59 months was conducted during November-December 2008 to provide national and regional estimates. A multi-stage cluster sampling method was applied and 40 gewogs/thromdes were selected from each region (Western, Central, Eastern). Guidelines on how to measure length/height and weight followed WHO standardized procedures. Data were analysed for consistency and validation using the software WHO Anthro and the WHO SPSS macro. Underweight, stunting, overweight, wasting and thinness were defined based on the WHO Child Growth Standards. Data from 1986-88 and 1999 national surveys were reanalysed using the WHO standards to describe trends in nutritional status.

**Results:**

Nationally, 34.9% Bhutanese preschool children are stunted and 10.4% are underweight. Wasting is 4.7%, with severe wasting close to 2% in rural areas, while overweight affects 4.4% of preschool children. While underweight rates are similar across regions, wasting is substantially more prevalent in the Western region and stunting in the Eastern region. Stunting shows a steep rise during the first two years of life, as high as 40%, and levels off thereafter, while wasting is greatest among children aged 6-24 months and subsequently decreases. The prevalence of stunting fell from 60.9% in 1986-88 to 34.9% in 2008, and underweight declined from 34.0% to 10.4% during same period. The percentage of wasted children dropped from 5.2% in 1986-88 to 2.5% in 1999 but then increased to 4.7% in 2008.

**Conclusions:**

There have been major improvements in the nutritional status of Bhutanese children over the past two decades, however, linear growth retardation remains a significant concern. Early identification of growth faltering is essential for improving the effectiveness of public health programs to prevent stunting. Similarly, wasting rates indicate the need for a system to identify children with severe malnutrition in the isolated communities so that they can receive appropriate care.

## Background

Located in the Himalayas, the Kingdom of Bhutan − or Druk Yul (Land of the Thunder Dragon) − is steadily opening up to the outside world and investing in human development while preserving its core traditions. Population distribution has been largely determined by the country’s geographical location and its rugged and steep terrain. This has obliged people to settle wherever they can find usable land, resulting in a scattered population where most people lack easy access to motor able roads.

Nevertheless, a committed government under the guidance of successive Kings has extended an impressive range of public services across this spectacular landscape, resulting in significant improvements in living conditions. Some of the most striking advances concern health. Between 1960 and 2005, life expectancy rose from 37 years to 66 years, while infant mortality fell from 203 to 40.1 deaths per thousand live births. These gains are primarily the result of basic preventive and curative health services - which now cover over 90% of the population - complemented by improved water supply and sanitation [1].

However, as Bhutan steadily proceeds along its development path substantial challenges remain, including malnutrition in young children with very high rates of stunting (47.7%) reported for children aged 6-59 months in the 1999 national nutrition survey [2]. Stunting (low length- or height-for-age) or poor linear growth is the result of multiple circumstances and determinants, including antenatal, intrauterine and postnatal malnutrition [3]. Stunting in early life is associated with adverse functional consequences, including poor cognition and educational performance, low adult wages, and lost productivity; and when accompanied by excessive weight gain later in childhood, increased risk of nutrition-related chronic diseases [4].

Bhutan's isolation from the rest of the world - until the establishment of the national airline in 1983 the country was almost entirely inaccessible - has resulted in few reports on the health and nutritional status of Bhutanese children. Morrow described the experiences of Thimphu General Hospital over a three-year period [5] in the first account of child health in Bhutan published in 1987. The objective of this paper is to contribute to this meagre body of evidence by reporting results from the National Nutrition and Infant and Young Child Feeding Survey (hereafter referred to as the survey) conducted in 2008 and describing progress in child nutritional status during the last two decades.

## Methods

The survey is a cross-sectional study designed to provide national and regional estimates. All of the country’s 20 Dzongkhags (districts) were grouped into three regions: Western (Thimphu, Paro, Punakha, Haa, Gasa, Chukha and Samtse), Central (Wangdue, Trongsa, Bumthang, Zhemgang, Tsirang, Dagana and Sarpang) and Eastern (Mongar, Lhuenste, Trashigang, Trashiyangtse, Pemagatsel and Samdrupjonkhar). A multi-stage cluster sampling method was applied and 40 gewogs/thromdes (geographic administrative units compose of several villages) were selected from each region using the Probability Proportional to Size (PPS) sampling technique. A minimum of three starting points were randomly selected within each gewog/thromde to increase the probability of being sampled within the gewog/thromde. Based on a stunting prevalence of 40% (from the 1999 anthropometric survey), precision of 5%, confidence interval of 95%, design effect of 2, and 5% of non-response rate, the target sample size came to 774 children for each region, or 2322 nationally.

Twenty survey teams of 3 members each (one supervisor and two field workers) were trained to administer the questionnaire and take anthropometric measurements. As part of their training, study teams were attached for two days to the Nutrition Rehabilitation Unit in the paediatric department of the National Referral Hospital, Thimphu, where they undertook visits to the nearby Basic Health Units.

Data collection took place during November-December 2008. After obtaining consent from the relevant district and gewog/thromde authorities to conduct the survey, the teams visited households to administer the questionnaire and take measurements. The questionnaire included 60 questions with modules concerning the mother, the index child, infant and young child feeding practices, sanitation and hygiene, and dietary history of the mother and index child. The training on anthropometric measurements was conducted by WHO-trained nutritionists following WHO standardized procedures [6]. The presence of a kitchen garden, the variety of vegetables grown, safe drinking water and latrines was noted in the questionnaire. Teams from the Ministry of Health and UNICEF monitored data collection.

The data entry tool was prepared using CSPro software, version 3.2. Data were entered in batches according to the gewogs/thromdes in a Dzongkhag for a maximum of 20 questionnaires each. Consistency checks and skip patterns were built into the entry program. Data cleaning and validation were done at several stages to ensure data errors were captured. All batches were merged and exported into SPSS for standardized analysis using the software WHO Anthro [7] and the WHO SPSS macro [8] for consistency and validation. Underweight, stunting, wasting and thinness were defined, respectively, as the proportion of children below -2 and below -3 (severe) standard deviations (SD) of the weight-for-age (WFA), length/height-for-age (LHFA), weight-for-length (WFLH), and body mass index-for-age (BMIFA) median of the WHO Child Growth Standards [9,10]. Overweight was defined as the proportion of children above +2 SD of the WFLH median of the WHO growth standards [9,10].

The survey received ethical clearance from the Research Ethics Board of Health and the National Statistical Bureau. Individual consent to participate in the survey was given by the child’s caregiver.

## Results

The survey covered 20 districts, 120 gewogs/thromdes and 501 villages. A total of 2376 mothers were interviewed and the anthropometric measurements of 2376 children aged 6 to 60 months were taken. The sample distribution by region was: 804 (33.8%) Western region, 798 (33.6%) Central region, and 774 (32.6%) Eastern region. Table 1 presents the number of children surveyed for each region. Complete information was available for a total of 2162 children, who were included in the analysis. The 214 interviews that could not be included in the analysis was due to incomplete dates of birth, implausible measurements, and missing weight and/or height.

**Table 1:** Number of children surveyed from each region

**Region**	**Boys**		**Girls**		**Both sex**	
	**Number**	**%**	**Number**	**%**	**Number**	**%**
Western	430	34.1	374	33.5	804	33.8
Central	420	33.3	378	33.9	798	33.6
Eastern	410	32.6	364	32.6	774	32.6
** Total**	1260	100	1116	100	2376	100

Western: Thimphu, Paro, Punakha, Haa, Gasa, Chukha and Samtse.

Central: Wangdue, Trongsa, Bumthang, Zhemgang, Tsirang, Dagana and Sarpang.

Eastern: Mongar, Lhuenste, Trashigang, Trashiyangtse, Pemagatsel and Samdrupjonkhar.

**Figure 1:**
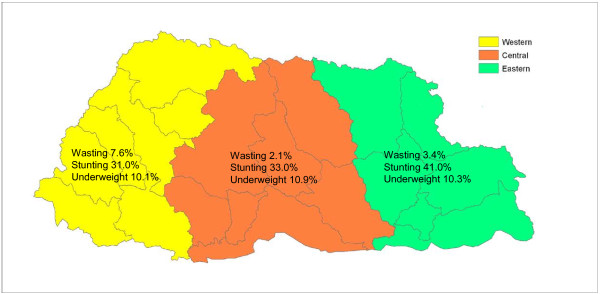
Prevalence of wasting, underweight and stunting by region, Bhutan 2008.

Figure 1 shows the prevalence of wasting, stunting, and underweight for Bhutanese children less than 5 years of age by geographical region. While the prevalence of underweight was quite similar across the three regions (about 10.4%), wasting was statistically significant more prevalent in the Western region (7.6%) − compared to 2.1% and 3.4% in the Central and Eastern regions, respectively − and stunting in the Eastern (41.0%) compared to the Western and Central regions (31.0% and 33.0%, respectively).

Table 2 shows the prevalence of underweight, stunting, wasting, thinness and overweight for Bhutanese children less than 5 years of age by sex, age group, and residence (urban/rural). Nationally, one out of three Bhutanese preschool children are stunted (34.9%), and 12% are severely stunted. Prevalence rates are somewhat higher in boys (37.7%) than girls (31.8%), and substantially higher in rural areas (37.1%) compared to urban areas (28.3%). About 10% of children nationwide are underweight, and less than 2% are severely underweight. Rates of underweight are similar in boys and girls, but substantially higher in rural versus urban areas (11.5% and 7.2%, respectively). The prevalence of wasting and thinness are similar, around 4.5%, with boys having higher rates than girls. The prevalence of severe wasting is quite high in rural areas (almost 2%) and in the Western region (2.7%). Overweight affects 4.4% of preschool children in Bhutan, with minimal variation by sex or region.

**Table 2:** Percentage of children under age 5 by nutritional status according to four anthropometric indicators, Bhutan 2008

	**Sample size**	**WFA % < -2SD**	**WFA % < -3SD**	**WFA mean Z-score**	**LHFA % < -2SD**	**LHFA % < -3SD**	**LHFA mean Z-score**	**WFLH % < -2SD**	**WFLH % < -3SD**	**WFLH % > +2SD**	**WFLH mean Z-score**	**BMIFA % < -2SD**	**BMIFA % < -3SD**	**BMIFA % > +2SD**	**BMIFA mean Z-score**
**Total**	2162	10.4	1.7	−0.70	34.9	12.0	−1.44	4.7	1.4	4.4	0.11	4.4	1.9	6.9	0.31
Male	1147	10.5	1.6	−0.74	37.7	11.9	−1.45	5.1	1.6	4.8	0.08	5.1	2.3	7.6	0.29
Female	1015	10.3	1.8	−0.66	31.8	12.1	−1.43	4.4	1.1	4.0	0.15	3.7	1.4	6.2	0.32
**Age:**															
6-11	202	5.4	0.5	−0.10	12.3	5.1	−0.31	7.1	3.3	2.7	−0.08	6.1	3.6	5.1	0.06
12-23	476	7.1	1.3	−0.38	26.3	10.8	−1.16	6.8	1.7	6.3	0.04	5.7	2.6	12.0	0.39
24-35	595	10.9	1.5	−0.75	38.1	12.9	−1.57	4.6	0.3	5.2	0.18	3.8	0.9	7.6	0.37
36-47	532	12.2	2.4	−0.94	42.1	12.2	−1.68	3.9	2.1	3.5	0.12	4.8	2.5	4.8	0.27
48-59	357	14.0	2.0	−1.04	42.9	15.6	−1.86	2.3	0.6	2.9	0.20	2.3	0.9	3.2	0.29
**Residence:**															
Urban	531	7.2	1.1	−0.53	28.3	7.4	−1.30	3.3	0.0	5.0	0.24	2.7	0.2	7.3	0.42
Rural	1631	11.5	1.8	−0.76	37.1	13.5	−1.49	5.2	1.9	4.2	0.07	5.0	2.5	6.8	0.27

WFA = Weight-for-age, LHFA = Length/Height-for-age, WFLH = Weight-for-length/height, BMIFA = Body mass index-for-age.

**Figure 2:**
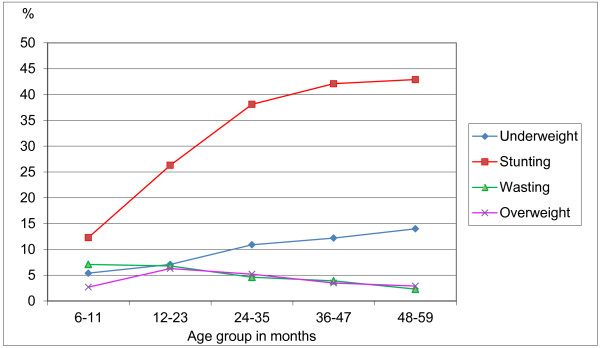
Percentage of children underweight, stunted, wasted and overweight by age group, Bhutan 2008.

The prevalence of stunting, underweight, wasting and overweight among children of different ages is shown in Figure 2. The pattern of stunting by age group shows a steep rise in prevalence during the first two years of life, as high as 40% and levelling off thereafter. The percentage of underweight children increases steadily with age, reaching 14% in those aged 48-59 months. The pattern of wasting by age group is quite different from that of stunting; the prevalence of wasting is greatest among children aged 6-24 months (about 7%) and decreases thereafter to reach the 2.3% expected in the standard population at 48-59 months. The low level of overweight (2.7%) in the 6-11 month age group increases to 6.3% at 12-23 months and thereafter follows a pattern similar to wasting.

**Figure 3:**
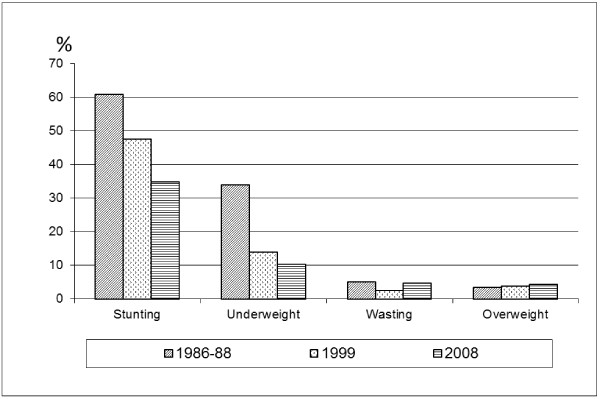
Prevalence of stunting, underweight, wasting and overweight in three national surveys (1986-88, 1999, 2008).

Trends in children’s nutritional status for the period 1986-88 to 2008 are presented in Figure 3. To allow the assessment of trends, data from the 1999 national survey were reanalysed using the WHO Child Growth Standards [11]. For the national survey conducted in 1986-88, the raw data were not available for reanalysis and the prevalences originally calculated based on the NCHS/WHO reference were converted into the WHO standards using an algorithm developed for this purpose [12]. Figure 3 shows that there have been major improvements in the nutritional status of Bhutanese children over the past 20 years. The prevalence of stunting fell from 60.9% in 1986-88 to 34.9% in 2008, and underweight rates declined from 34.0% in 1986-88 to 10.4% in 2008. The percentage of wasted children dropped from 5.2% in 1986-88 to 2.5% in 1999 but then increased to 4.7% in 2008. Overweight presents low levels that are steadily increasing (3.5% in 1986-88 versus 4.4% in 2008). The trends over time are statistically significant for stunting and underweight but not for wasting and overweight.

## Discussion

Child malnutrition remains one of the main public health challenges of the 21^st^ century. Recent global estimates suggest that stunting, wasting, and intrauterine growth retardation are responsible for 2.2 million deaths and 21% of disability-adjusted life-years lost among children under 5 [13]. Results from the third National Nutrition Survey in Bhutan show that while there has been a major decrease in the prevalence of stunting among children 6 months to 5 years (from 60.9% in 1986-88 to 34.9% in 2008), linear growth retardation remains a significant public health problem that poses a threat to the healthy growth and development of Bhutanese children. Compared to other countries, current stunting rates in Bhutan are categorized as high (range 30-39.9%) [14].

The problem of stunting starts early. As shown in Figure 2, the critical period for stunting in Bhutanese children is the first 2 years of life. This is consistent with child growth patterns worldwide [15] and confirms the importance of the first two years of life as a critical period when linear growth is most sensitive to environmentally modifiable factors. It also highlights the need for prenatal and early life interventions to avert the growth failure that occurs during this sensitive period and decrease the risk of maternal mortality and disability due to obstructed labour.

However, stunting often goes unrecognized, especially in communities where short stature is so common that it seems normal. Even among health workers, stunting generally does not receive the same attention as underweight or wasting, especially if height is not routinely measured as part of community health programs. Thus, in Bhutan as elsewhere, early identification of linear growth faltering is essential for improving the effectiveness of public health programs in preventing stunting. Likewise, the regional differences in the distribution of childhood stunting shown in Figure 1 need to be taken into consideration when planning interventions to tackle this problem. A separate survey should be carried out on a sub-regional level, like district, constituency or gewogs, with more sectors involved to provide information on all major factors related to undernutrition and investigate the underlying causes for the regional differences.

Rates of childhood underweight have dropped more than two-thirds since the first national survey (34% in 1986-88 versus 10.4% in 2008). Underweight is evenly distributed across geographical regions, with a national rate categorized as of moderate public health significance when compared with the overall estimate for Asia (19.5% in 2010) [16]. Of greater concern, however, is the prevalence of wasting (4.7% based on the 2008 NNS and 5.9% based on the MICS 2010 [17]), with prevalence rates up to 7% or more in the 6 to 24 month age group and the Western region (Table 2, Figure 1). These wasting rates indicate that acute undernutrition remains a matter of concern in early childhood, especially as severely malnourished children have 900 times higher risk of death [13]. Growth monitoring in Bhutan is carried out by health workers in the Basic Health Units (BHUs); however, children living in remote areas have little contact with BHUs, mainly at the time of immunization (at weeks 6, 10 and 14, and 9 and 24 months). An approach to identify these children in need of immediate attention will be to establish a system for identifying children with severe malnutrition in the community so that they can be referred to the BHUs for treatment. The screening for severely malnourished children could be done by village health workers using MUAC (assessments to be done on a monthly basis during the first year of life and every 3 months thereafter). MUAC should be assessed using a coloured tape (easy to interpret) following the WHO/UNICEF guidelines on the assessment of severe malnutrition [18]. Children identified as severely malnourished on the basis of low MUAC should be immediately referred to the BHUs where their weight-for-height should be assessed. It is important to note that the two indicators (MUAC and weight-for-height) do not always coincide [18], and therefore all children screened as severely malnourished on the basis of MUAC should be accepted and referred to the nearest hospital even if their weight-for-height is not below -3SD.

The high rate of stunting coupled with the moderate level of underweight indicates that childhood overweight is likely becoming a condition of concern in Bhutan. Although the rates are still low (4.4% of children aged 6-59 months were above +2SD of the WHO standards' weight-for-height), a slight but steady increase is observed across time (Figure 3).

Using the indicator BMI-for-age (an alternate indicator to assess childhood overweight) the national prevalence was 6.9%, with rates as high as 11% in the Eastern region [17]. These levels of childhood overweight are similar to the overall estimate for Asia in 2010 [19]. The high prevalence of both stunting and severe wasting, coupled with increasing rates of overweight, highlight the fact that monitoring weight-for-age alone is insufficient; it is also necessary to include the measurement of length/height to be able to monitor low height-for-age (stunting) as well as low and high weight-for-height (wasting and overweight) at both individual and population levels. Bhutan’s Maternal & Child Health (MCH) handbook is being revised to include sex-specific length/height-for-age charts based on the WHO Child Growth Standards.

Various factors are responsible for the decline in growth indicators, among them inadequate quantity and/or quality of complementary feeding relative to children’s energy and nutrient needs, or that serves as a channel for infectious agents and toxins. There is a large body of scientific evidence on what constitutes appropriate infant and young child feeding, from exclusive breastfeeding during the first 6 months of life [20] to guiding principles on complementary feeding [21]. More importantly, there is ample evidence that appropriate infant feeding practices result in better growth for infants and young children in poor environments. A recent analysis of Demographic and Health Survey data from 46 countries found that countries with low rates of exclusive breastfeeding and inadequate dietary diversity consistently had a high prevalence of undernutrition [16].

Bhutan’s Ministry of Health recommends exclusive breastfeeding from birth to 6 months of age. According to the 2008 survey, initiation of breastfeeding within the first hour after birth is high (81.5%) and the median duration of breastfeeding is 23 months. However, 14% of mothers introduce other foods (usually butter) by the first month, and by 6 months only 10% are exclusively breastfeeding their babies. Exclusive breastfeeding for 6 months is also challenging for mothers working in the public sector since they are granted only 3 months maternity leave while workers in the private sector have only 2 months.

The National Assembly has begun to discuss these issues, including proposals for a maternity support fund to provide minimum wages for 6 months to enable mothers to breastfeed. In addition to actions aimed at protecting and promoting exclusive breastfeeding for the first 6 months of life, it will be important to revise the feeding recommendations in the MCH handbook to include more specific information regarding both types and amounts of food and feeding frequency appropriate for each age group. The inclusion of Infant and Young Child Feeding indicators [22] in the Health Management Information System will strengthen the nutrition surveillance system and help monitor the adequacy of feeding practices for Bhutanese children. Similarly, future nutrition surveys should incorporate questions about childhood illnesses – especially diarrhoeal diseases and respiratory infections – given their impact on child growth trajectories.

## Conclusions

There have been major improvements in the nutritional status of Bhutanese children over the past two decades, however, linear growth retardation remains a significant public health problem. Early identification of growth faltering is essential for improving the effectiveness of public health programs to prevent stunting. Similarly, wasting rates indicate the need for a system to identify children with severe malnutrition in the isolated communities so that they can receive appropriate care.

## Abbreviations

BHUs: Basic Health Units; BMIFA: Body mass index-for-age; LHFA: Length/height-for-age; NCHS: National Center for Health Statistics; SD: Standard deviation; WHO: World Health Organization; WFA: Weight-for-age; WFLH: Weight-for-length/height.

## Competing interests

The authors declare that they have no competing interests.

## Authors’ contributions

UZ and TD participated in the design of the study, implemented the survey and revised critically various drafts of the manuscript. MdO participated in the design of the study, guided the analysis and interpretation of data, and drafted the manuscript. All authors read and approved the final manuscript.

## Pre-publication history

The pre-publication history for this paper can be accessed here:

http://www.biomedcentral.com/1471-2431/12/151/prepub
